# The Inheritance of Insanity

**Published:** 1908-08-15

**Authors:** 


### The Inheritance of Insanity.

There recently appeared in these columns a leading article entitled "The Pathological Aspect of Primogeniture," in which reference was made to the work done by Mr. Heron, of the Eugenics Laboratory. The paper there referred to is the memoir, "A First Study of the Statistics of Insanity, and the Inheritance of the Insane Diathesis"; and we should like to bring to notice one or two other points contained therein. In his introductory remarks, Mr. Heron rightly lays stress on the necessity, for the purpose of statistical study of this and kindred problems, of having a large collection of family histories, completely and scientifically recorded by those who have the opportunity and clinical training necessary for accurate observation. These histories are needed of normal as well as of tainted stock, as, without the former the latter are of little value. The work of obtaining complete family histories is dull and seems to be without result; but each one properly recorded will be of value to future generations. Mr. Heron compares the work to that of the astronomer "who piles up data that his successor a century hence may, by comparing the positions of stars at distant intervals, learn the truth." With respect to the fact referred to in the former article that insanity seems to appear more frequently amongst the earlier than amongst the later born members of a family, it is to be noted that insanity occurring at or after adult age is being studied; thus cases of idiocy and imbecility due to injury to the head in earlier confinements are excluded. It appears probable, also, that inheritance of the insane diathesis follows the orderly system of inheritance in man of other parental characters, and is therefore capable of elimination by prudential mating in successive generations. The last point to be noted, and that which probably more than the others causes one to think deeply, is that stocks tainted with mental instability like deaf-mutism, tuberculosis, criminality, etc., are (as found by Pearson, not less, but rather more, fertile than normal stocks. Near the conclusion of the memoir occur the following sentences, which deserve quotation at length: "The normal, or probably more than normal fertility of the insane, notwithstanding their partial seclusion and high death-rate, and the fact that insanity is a disease largely of middle life, must make us pause before we acquiesce in the unrestricted return of the 'recovered' insane to family life; while the definite inheritance of insanity which is here demonstrated, an inheritance as intense as that of deaf-mutism or the tubercular diathesis, must surely raise the question of the morality of the marriage, and, above all, of the intermarriage of members of insane stocks. The time appears to be rapidly approaching when society, to protect itself, shall check by even more than moral condemnation the free marriage and unrestricted fertility of those of tainted stock."

